# Understanding fathers’ perinatal mental health and well-being support needs: a qualitative study of fathers’ and professionals’ perspectives in North East England and North Cumbria, UK

**DOI:** 10.1136/bmjopen-2026-121953

**Published:** 2026-09-04

**Authors:** Ashleigh Elizabeth Watkins, Catherine El Zerbi, Ruth McGovern, Judith Rankin

**Affiliations:** 1 Population Health Sciences, Newcastle University, Newcastle upon Tyne, UK; 2 Division of Health Sciences, Lancaster University Faculty of Health and Medicine, Lancaster, UK

**Keywords:** MENTAL HEALTH, Postpartum Period, Health Services

## Abstract

**Abstract:**

**Objectives:**

The transition into fatherhood is considered a profound life stage, involving personal development, lifestyle and emotional adjustments. Fathers’ mental health can be adversely impacted by this transition. Fathers express isolation, exclusion and limited support within perinatal services. Restricted emotional support for fathers presents negative consequences for the whole family dynamic. Limited research has explored father and professional input associated with paternal perinatal support and how healthcare services and child and family services can respond to engaging fathers and their mental and emotional support needs. This qualitative study aims to explore fathers’ mental health and well-being experiences (referring to emotional or/and social well-being) and support needs within the perinatal period, as well as current viewpoints of perinatal services (maternity, healthcare and social care services) from both fathers’ and professionals’ perspectives.

**Design:**

In-depth semistructured interviews and focus groups were carried out as part of a qualitative study.

**Setting:**

Fathers’ resident within the North East and North Cumbria (NENC) and services and organisations from the voluntary, community and social enterprise (VCSE) sector and social care services across the NENC, who associate with supporting families and children, and perinatal mental health.

**Participants:**

Fathers (n=21) and professionals (VCSE and social care services) (n=9).

**Results:**

Reflexive thematic analysis of 30 participants’ accounts identified two main themes and five subthemes: (1) ‘The Pregnant and Postnatal Man’ and (2) Removing the cloak of fatherhood invisibility. These themes centred around the isolation of fathers and limited emotional and mental support within the parenting transition.

**Conclusions:**

The findings suggest that greater father inclusion within perinatal services, policies and antenatal education may help fathers feel more recognised and supported throughout the perinatal journey. Recognition of fathers’ emotional well-being and mental health, by adopting a ‘whole family’ emotional support approach within perinatal services was considered important to support fathers and the family dynamic in facilitating a positive transition for the whole family.

**STRENGTHS AND LIMITATIONS OF THIS STUDY**

- Limited research has explored professional inputs, especially those from the voluntary, community and social enterprise (VCSE) and social care sectors; thus, this study explores both fathers from differing backgrounds and ethnicities and professionals’ accounts of perinatal services (maternity, healthcare and social care services).
- Recruitment of adoptive fathers, and specifically fathers from low-income backgrounds, was limited.
- Although many participants from the VCSE sector had previously/currently worked within the National Health Service (NHS) or healthcare settings, no health professionals were recruited directly through the NHS.

## Background

 While often a very joyous and celebratory time, the transition into parenthood is considered a profound life transition with many emotional adaptations and radical lifestyle changes with work, financial stability, social support and personal development.^1–3^ The perinatal period is defined as the time period from conception (antenatal) up to the first 12 months after childbirth (postnatal).^4^ Within the postnatal period, particularly many parents, such as fathers, have expressed heightened depressive symptoms, poor mental health and distress due to lack of sleep, not feeling supported, going back to work when not ready or having to take on extra responsibilities.^5–8^ Over recent decades, expectations of fatherhood have evolved, with fathers increasingly expected to combine the traditional role of financial provider with active involvement in caregiving, emotional support and nurturing of their children.^2^ In many high-income countries, this shift has been accompanied by greater paternal involvement during pregnancy, childbirth and early parenting, reflecting changing social norms surrounding fatherhood and masculinity.^3^ However, balancing these multiple and sometimes competing expectations can create tensions as men negotiate their emerging identities as fathers alongside employment, partner relationships and other responsibilities. These challenges may influence fathers’ psychological well-being and their transition to parenthood.^2 9^ Previous research exploring the association of the transition to fatherhood and mental health and well-being has been limited, although recent evidence has indicated that men’s mental health can be adversely impacted by this transition.^10^

Although woman-centred maternity care remains fundamental, there is increasing recognition that fathers should also be acknowledged and supported as part of family-centred perinatal care.^11^ However, evidence suggests that health professionals often face organisational and structural barriers to engaging fathers, including service models, institutional practices and gendered assumptions that continue to position mothers as the primary recipients of care.^12 13^ These challenges may also contribute to difficulties in engaging partners and ensuring that perinatal services are inclusive of diverse family structures, including same-sex couples.^14^ Tarrant^11^ further argues that institutional policies, professional language and service practices can systematically marginalise fathers as caregivers, limiting opportunities for meaningful involvement during the perinatal period. These systemic barriers may be reinforced by the limited availability of father-inclusive training for healthcare professionals, restricting their confidence and capacity to engage fathers effectively.^15^ Previous studies have also highlighted that professionals’ attitudes and behaviours have contributed to many fathers feeling excluded throughout their perinatal journey, including the distinct lack of mental health support for men as they transition into fatherhood.^16–19^ Prior research has explored how healthcare services and child and family services can respond to engaging fathers and their support needs by promoting father-inclusive practice from professionals and within services by proactively engaging fathers, challenging stereotypes and biases and providing education and support.^20–22^ However, existing research does not specifically explore engagement strategies associated with fathers’ mental health and well-being within the perinatal period; instead, it offers a more generalised approach to engagement within the perinatal period.

Within the past 5 years, the North East and North Cumbria have had the highest rates of male suicides across the UK.^23^ Prior research has identified that fathers are up to 47 times more likely to be identified as a suicide risk in the perinatal period than at any other point in their lives. According to a recent 2025 report on Paternal Suicide in the 1001 Critical Days, two to three babies lose their fathers to suicide every week across the UK.^24 25^ These statistics highlight the importance of exploration around fathers’ mental health in the perinatal period, specifically within the North East and North Cumbria (NENC). Past systematic reviews have recognised that fathers under-represented of all backgrounds (biological and adoptive/minority ethnicities) support needs and mental health and well-being experiences across qualitative research.^16–18 26^ Recent research by Tarrant, Way and Ladlow^27^ highlights the importance of adopting an intersectional approach to father-inclusive care, recognising that fathers’ experiences and support needs are shaped by intersecting social, cultural and structural factors. The authors argue that healthcare professionals should tailor engagement and support to the diverse circumstances of fathers, including addressing structural inequalities and strengthening culturally responsive practice. Further research is needed to understand the challenges and support needs of all parenting transitions, through inclusivity of fathers from diverse ethnicities, of various ages and differing family dynamics, to address needs and inequalities within the implementation of support across the NENC.^16–18^

An increasing body of research has evaluated father-inclusive antenatal programmes and interventions designed to support fathers during the transition to parenthood.^28 29^ However, much of this evidence is derived from quantitative studies, with comparatively less research exploring fathers’ experiences, support needs and perspectives on how perinatal services can better engage and support them. Future research recommendations also included further exploration of subjective experiences of attending antenatal programmes and support within perinatal services.^4 17 30^ Past research is limited when exploring professional input associated with paternal perinatal support and service development. Therefore, exploration with professionals of different backgrounds, perspectives and viewpoints on inclusion and fatherhood engagement within emotional and mental health support is required across health and social care services and voluntary, community and social enterprise (VCSE) organisations (providing support in the perinatal period, NENC) to inform future practice and policy implications.^21^

This qualitative study aims to explore fathers’ mental health and well-being experiences (referring to emotional or/and social well-being) and support needs within the perinatal period, as well as current viewpoints of perinatal services (maternity, healthcare and social care services) from both fathers’ and professionals’ perspectives. The specific objectives were to: (a) explore fathers’ resident within the NENC lived mental health and well-being experiences within the perinatal period and objective, and (b) develop an understanding of fathers’ support needs and how these might be addressed within perinatal services from both fathers’ and professionals’ perspectives (NENC), laying the foundations for future development, design and recommendations for perinatal public health.

## Methods

### Setting

The study was conducted at Newcastle University in Newcastle-upon-Tyne, UK, as part of a funded PhD with the National Institute for Health Care Research (NIHR) Applied Research Collaboration (ARC) NENC. Data collection was conducted online. The research was undertaken within the NENC, a region characterised by high levels of socioeconomic deprivation and the highest suicide rates in England.^31^ Despite increasing recognition of paternal mental health during the perinatal period, regional data on paternal perinatal mental health remain limited, highlighting an important evidence gap within the NENC.^25 32^ Fathers from diverse backgrounds residing within the NENC were invited to participate to explore their experiences of perinatal mental health, well-being and support. To complement these perspectives, professionals from VCSE organisations and social care services across the NENC that support families, children and perinatal or paternal mental health were also recruited. Including both fathers’ and professionals’ perspectives enabled exploration of individual experiences alongside the organisational context in which perinatal support is delivered.

### Study design

A qualitative research design was employed to explore fathers’ mental health and well-being experiences and support needs within the perinatal period, as well as current viewpoints of perinatal services (maternity, healthcare and social care services) from both fathers’ and professionals’ perspectives. A semistructured interview method was used with fathers and professionals; verbal interchanges between the interviewer and participant, adopting a flexible open-ended questioning approach.^31 32^ Alternatively, professionals had the option to participate in a focus group with other colleagues instead of an interview; focus groups are a group of people who talk about a set topic guided by the researcher.^32^ This allowed for interactions between work colleagues and changing perspectives.^33^ The topic guide (see online supplemental file 1) was approved by Newcastle University’s Medical Ethics Committee (see ethics section below). Informed consent was obtained from all participants electronically before the date of the interview; participants were made aware they could withdraw at any point and fully debriefed after the interview/focus groups. Fathers (who had a child in the past 3 years) and professionals (associating with family support and perinatal/paternal mental health support) were purposely selected. To assist with the reporting of this study, the Consolidated Criteria for Reporting Qualitative Research (COREQ): a 32-item checklist for interviews and focus groups, was followed to ensure important aspects of the research were included.^34^

### Recruitment and procedure

Recruitment comprised ‘Purposive sampling’ and ‘Snowball sampling’ to achieve maximum variation of the samples (fathers and professionals) and reach networks potentially missed. Two poster advertisements detailing the study to both fathers and professionals were created by AW. Participants were recruited via contact with fathers who were part of the patient and public involvement (PPI) group within the PhD project, as well as fathers who had previously shown interest in participating in the project but preferred to take part in the qualitative study rather than PPI workshops (n=7). Fathers were advised to reach out to friends or work colleagues who may be interested. Contact was also made with professionals previously networked with throughout the PhD project (emails and meetings) (n=5). Participants were also recruited through advertisement of the two posters on the NIHR ARC NENC website and newsletter (n=5) and via a range of health, social and community services within the NENC (n=13). Potential participants were asked to email AW if they were interested in taking part. Father participants were sent an additional link to an online Qualtrics survey to fill out an expression of interest. The survey included demographic questions to assess eligibility. Table 1 presents the inclusion and exclusion criteria for both fathers and professionals. Father and professional participants who met the inclusion criteria were identified and contacted for participation in the study. Recruitment took place between April and September 2023.

**Table 1:** Inclusion and exclusion criteria of participants

	Inclusion criteria	Exclusion criteria
Fathers	Fathers (an individual who has parental responsibility of a child, this can include biological, adoptive fathers or stepfathers) Aged 18+ No restrictions relate to first-time or experienced father, age or marital status Based in NENC, UK Child between 0 and 3 years old	Young fathers under the age of 18 Fathers who are not based within the NENC, UK Fathers who do not speak English Fathers who have a child over the age of 3 years old Fathers who do not have access to online platforms.
Professionals	VCSE volunteer/employee Social care services practitioner/social care worker	Professionals in roles that are not associated with supporting (direct or indirect involvement) fathers ‘or families’ mental health or perinatal mental health in the NENC.

NENC, North East and North Cumbria; VCSE, voluntary, community and social enterprise.

A participant information sheet tailored to either a father or a professional participant was sent by AW. The information sheet also stressed that taking part was entirely voluntary and that they had the right to stop the interview at any given point. If the participant agreed to continue with the study, an electronic informed consent sheet was also sent and asked to sign prior to the interview/focus group. Interviews did not commence without prior informed consent. After the interview, participants were provided with a debrief sheet signposting participants to relevant support services if required. Participants were allocated a £25 voucher for their time.

### Data collection

Data collection took place from May to September 2023. Semistructured interviews with fathers (n=21) and semistructured interviews or focus groups with professionals (n=9) were conducted simultaneously. Two focus groups were conducted with professionals, one with the VCSE and one with social care services. All of the interviews and focus groups were conducted by AW and were audio or video recorded for transcription purposes. Father interviews ranged in length from 23 to 68 min, and professionals’ interviews or focus groups ranged from 41 to 65 min. All interviews and focus groups were conducted online using the online platforms Zoom or Teams. No interviews or focus groups were conducted face-to-face; this was due to participant preference.

Interviews/focus groups started by asking participants a series of demographic questions; father participants were asked two overarching questions focusing on experiences transitioning into fatherhood (emotional and social experiences). Followed by focusing on experiences of support in the perinatal period (prenatal and postnatal). Similarly to professionals, interviews/focus groups were also split up into two sections: experiences of professional roles associated with fathers within the perinatal period and opinions and perspectives of current paternal perinatal support. These topic guides were revised after consultation with co-authors (JR, RM and CEZ) and piloted by AW with one PPI father prior to data collection. The semistructured approach enabled flexibility throughout data collection, allowing participants to elaborate on additional information not covered in the topic guide. Interviews/focus groups were transcribed using the online platforms Zoom and Microsoft Teams.

Demographic data, including gender, age, ethnicity, employment status, educational background and previous mental health diagnoses, were collected from participants depending on whether they were a father or a professional. All interview transcripts and personal data were anonymised using pseudonyms that were also employed when reporting the data. All data were stored securely on a private, password-protected or encrypted server managed by Newcastle University, only accessible to the research team.

### Data analysis

The analytical approach employed within this research study was reflexive thematic analysis due to its appropriateness with this study’s philosophical orientation (see reflexivity and philosophical orientation below). There are six initial phases within the Braun and Clarke thematic analysis approach: familiarisation with the data, coding, searching for themes, reviewing and finally naming themes.^35 36^ The analysis reports on an empirical exploration of fathers’ mental health and well-being experiences within the perinatal period, their support needs, as well as fathers’ and professionals’ perspectives of current perinatal services and design in the NENC (healthcare, maternity and social care services). Data analysis was conducted by AW, with co-authors double coding up to 25% of transcripts (JR, RM=10% & CEZ=25%) on NVivo qualitative analysis software. Regular data meetings with co-authors (JR, RM and CEZ) were conducted to discuss personal interpretations, allowing for multiple assumptions and interpretations of the data from differing personal experiences. Two data meetings with AW and CEZ were conducted to discuss important concepts and theme development. Final themes and subthemes were agreed with all co-authors and titled using short illustrative quotes followed by an analytical take on the theme.^37^ The presentation and narrative of the data combined both father and professional perspectives to elicit exploration of comparisons and contradictions of concepts within themes.

### Reflexivity and philosophical orientation

The philosophical orientation of critical realism was used for this study to recognise the underlying mechanisms and influences within the transition to fatherhood, on mental health impacts/experiences and beneficial support for fathers in the perinatal period. Critical realism allows more of an explanation analysis as opposed to empirical description with comparison among participants’ accounts that can contribute to the formation of conclusions of participants’ experiences and suggestive solutions for social change.^38^ The approach can therefore also contribute to knowledge in a meaningful way, specifically when relating to healthcare research.^39 40^ A qualitative primary exploration and methods and analytical approach (reflexive thematic analysis) were chosen for this research to align with this philosophy. Within critical realism, data are informative of reality, but do not mirror it, and therefore need to be interpreted, allowing interaction with the data and access to underlying structures.^41^ Themes within reflexive thematic analysis can be developed that are symbolic of differing levels of interpretation, capturing ‘surface level’ data, as well as ‘hidden meaning’ and conceptualisations discovered within the data, aiding beneficial understandings and recommendations for practice and policy to inform public health. A combination of both semantic (surface level) communicated by the participant and latent (hidden meaning/interpretation from the researcher) coding was captured within the analysis.

Critical realism recognises the importance of reflexivity from the researcher due to interaction with the data, bias can be formed through differing backgrounds and contexts. It was important to recognise within this study that the first author (AW) identifies as a white British woman in her late 20s, with no children, who had previous experience in perinatal mental health clinical and research settings. In comparison, participants across this research consisted primarily of men, while exploring their mental health within the transition to fatherhood. Reflective accounts of the interviewing process showed that due to the heightened associated taboo, fathers required time to build a trusting and engaging relationship before disclosing experiences. However, overall, men favoured being more emotionally vulnerable and comfortable with a woman expressing mental health and well-being difficulties.

### Patient and public involvement (PPI)

The design of this study and research objectives were informed by a previous scoping review^17^ and gaps in findings that were generated with support from a PPI group of fathers. Fathers who were part of this PPI group throughout the PhD project supported the development of ethics documentation for this study, including the participant information and debrief sheets.

### Ethical considerations

Ethical approval was obtained from the Newcastle University Ethics Committee (12 April 2023; Ref 2505/30066/2021). It was possible that participants may have experienced distressing events or have found it distressing to talk about mental health. A risk management document was developed before data collection, acknowledging all potential risks and the appropriate procedures put in place. A full debrief was provided at the end of the study, signposting participants to support services.

## Results

### Fathers’ and professionals’ sample characteristics

In total, 30 participants took part, fathers (n=21) and professionals (n=9). Table 2 presents the demographics of all participants.

**Table 2:** Participant demographics

Participant demographics	Fathers	Professionals
Gender		
Male	21	3
Female	–	6
Age		
18–25	3	0
26–35	12	4
36–45	6	3
45	0	2
Ethnicity		
White British	12	8
Black British	4	0
Black African	3	0
Black Caribbean	1	0
Asian	1	0
White Black Caribbean	0	1
Residence		
Newcastle	8	–
Morpeth	1	–
Whitley Bay	1	–
Sunderland	3	–
Washington	1	–
Consett	1	–
Darlington	1	–
Middlesbrough	2	–
Stockton-on-tees	3	–
Employment status		
Employed full time	11	–
Employed part time	5	–
Student	3	–
Unemployed	2	–
Time period		
0–1-year-old	5	–
1–2 years old	7	–
2–3 years old	9	–
Father		
Experienced father	7	–
First-time father	14	–
Relationship status		
In relationship and co-habiting with mother of child	21	–
Not in relationship or co-habiting with mother of child	0	–
Mental Health diagnosis		
Yes	9	–
No	12	–
Organisation/association		
Children’s social care services	–	5
VCSE		4

Demographic statistics of participants (fathers n=21) and professionals (n=9) total (n=30).

VCSE, voluntary, community and social enterprise.

Reflexive thematic analysis of 30 participants’ accounts identified two main themes and five subthemes associated with objectives one and two: (1) ‘The Pregnant and Postnatal Man’ and (2) Removing the cloak of fatherhood invisibility. Table 3 presents the themes and subthemes.

**Table 3:** Thematic themes and subthemes

Theme	Subthemes
Theme 1: ‘The Pregnant and Postnatal Man’	Paternal mental transitional challenges (Objective 1) Physical pregnancy absence (Objective 1)
Theme 2: Removing the cloak of fatherhood invisibility	Encompassing a whole family emotional support approach (Objectives 1 & 2) “What is antenatal information?” Strive for inclusion and equity of antenatal resources (Objective 2) *“*A system furnished for mum” Development of service infrastructure and policies targeting maternal caregiver bias (Objective 2)

Thematic themes and subthemes and addressed objectives for each theme.

### Theme 1: ‘The pregnant and postnatal man’

This theme included two subthemes: Paternal transitional mental challenges and Physical pregnancy absence.

### Sub-theme 1: Paternal transitional mental challenges

All fathers expressed substantial transitional and mental challenges throughout the perinatal period; these challenges encompassed anxieties, fears, low mood, economic struggles, social exclusion and confrontation from friends and family. Fathers reported a responsibility shift, whereby they had to stabilise multiple roles, including their work (employee), family (father and husband) and focus on their own well-being and mental health within the postnatal period. The navigation of being a parent was referred to as “unpredictable” and “the biggest challenge” (Alfie, Father, early 30s) due to the extra reliance from a child. Another father stated, “the first few weeks were hell” (Ross, Father, late 30s). Other fathers also reflected poor mental health and low mood associated with the loss of self-identity:

You’ve still got to find that balance between being a dad and still being yourself, because I lost myself somewhere in that process and I’m only just now starting to find myself. (Dale, Father, early 40s).

Accounts from adoptive fathers highlighted supplemental stresses and emotional expectations of transitioning into fatherhood. This was considered due to the child’s additional needs and “therapeutic parenting approach” (Alfie, Father, early 30s). They expressed, similar to biological fathers, aspirations and gratefulness to becoming a father, viewing children “as a blessing” (Aaron, Father, mid 20s). This was particularly apparent among fathers of minority ethnicities and religious backgrounds/values. These fathers stressed more so the importance of the family dynamic, with ingrained cultural values and acceptance that the male identity is underpinned by husband and fatherhood responsibilities. However, adoptive fathers also recognised a decline in mental health postnatally, potentially due to expectations of fatherhood not always aligning with reality. This was referred to as “kind of post-adoption depression syndrome” (Alfie, Father, early 30s) by an adoptive father who felt particularly impacted by the challenges within the transition.

Accounts from fathers with low socioeconomic status, particularly young fathers from low-income backgrounds, highlighted financial pressures and stresses that resulted in worsening their mental health: *“*financially I wasn’t like that stable, I wasn’t ready” (Finn, Father, mid 20s). Many of these fathers were unemployed or working part-time hours, experiencing socioeconomic challenges and restrictions, contributing to more negative transitions.

Many fathers also discussed contradictory parenting styles and advice that led to “heated debates” (Conor, Father, mid 30s). Cultural differences between families were also seen to create disputes and disagreements due to different cultural expectations on parenting practices. For example, ‘British vs African culture’: “my parents expected my son to grow up in an African culture” (Kwame, Father, late 20s), initiating emotional barriers and challenges.

Despite the shared negative mental health experiences and emotional and physical exhaustion, some fathers did still acknowledge the positivity and growth in themselves as a result of becoming a father: “my self-esteem was boosted, I felt like I’m now a man” (Zac, Father, mid 20s), “they were not a distraction, I was happy” (Luke, Father, mid 30s).

#### Subtheme 2: Physical pregnancy absence

The reality of becoming a father was sometimes considered hindered by the absence of physically experiencing pregnancy. Fathers expressed detached viewpoints and experiences throughout pregnancy, such as “it got as real as it was going to get for me before the birth” (Danny, Father, early 30s). Fathers often struggled to imagine their future child physically, delaying the mental transition. They talked about a lack of maternal instinct and bond that they assumed was ingrained in the biological and physical experience of mothers’ pregnancy. It was pointed out by fathers that the mental transition to fatherhood was accelerated through attendance of ultrasound scans, by igniting connection and encouraging future bonding experiences after birth, which had positively enhanced their paternal experience:

“It (attending ultrasound scan) really helped, because my mental transition started from there it helped with the bonding straight away.” (Conor, Father, mid 30s)

On the other hand, a few fathers did not express the importance of attending appointments for their own psychological means, in building a connection or helping the progression of the transition, but as more of a supportive figure: “I don’t think for the appointment itself I don’t think it was important for me.” (James, Father, early 40s).

An adoptive father highlighted the thorough and intrusive process involved when adopting children, as opposed to the pregnancy experience. The absence of pregnancy meant that adoptive fathers did not experience ultrasound scans that could potentially enhance their paternal experience. Similar to biological fathers, adoptive fathers expressed that the initial reality of becoming a father was only apparent once they had their children placed with them on *‘*day one’ (Alfie, Father, early 30s).

Most fathers stated they took on a more practical and resourceful role to rationally fulfil the fatherhood figure and the role of supporting their partner. This was a way to compensate for not being physically pregnant and able to offer maternal support. The fathers highlighted more negative connotations around breastfeeding, viewing it as an impediment to bond development: “One thing that has been difficult is for my well-being is not being able to kind of do something more practical for the breastfeeding.” (Michael, Father, early 30s). They emphasised the importance of opportunities to bottle feed, tasks that were not entirely associated with maternal dependency. The absence of physical pregnancy was also recognised by practitioners as impactful on fathers, acknowledging the need for postnatal tasks that could be experienced together to help bond development and mental transition:

“Dads haven’t had that bond in that they haven’t grown a child, I think it’s that this is this is joint. (Jess, SCS, Project Development Officer)

### Theme 2: Removing the cloak of fatherhood invisibility

#### Subtheme 1: Encompassing a whole family emotional support approach

Despite the difficulties outlined above, fathers from diverse backgrounds consistently reported that they were not offered support by services to the same extent as their partners. It was expressed by both fathers and practitioners throughout that there was a greater focus on the “woman’s needs” rather than a “family approach” (Phoebe, VCSE, Operational Employee) to parenting support within healthcare and social care services.

All fathers respected that their partners’ needs were the priority. However, fathers whose partners had been diagnosed with perinatal mental health (PMH) disorders, including postnatal depression, post-traumatic stress disorder (PTSD) and bipolar disorder or required additional support. These fathers found supporting their partner exceptionally challenging without professional, practical or mental support for themselves. One father, Paul, expressed this when his wife was “unable to cope in a normal maternal manner; I had to fill that void and be father and mother; it was more demanding” (Paul, Father, late 30s)*,* increasing his stress and affecting his well-being. These fathers felt professionals addressed primarily the support needs of their partner, which often left little recognition or understanding of fathers’ viewpoints or struggles. Many of these fathers discussed the non-acknowledgement of their emotional well-being; they desired no mental health specialised support, but a conscious understanding from professionals of the heightened expectations on the father. This awareness also needed to be relayed to their partner, to highlight their perspective and contribute to more effective communication and understanding in the relationship:

“I didn’t feel like they explained stuff to my partner to help understand me” (Adam, Father, mid 20s).

The majority of fathers stressed the need for professionals across healthcare, maternity services and social care to be inclusive of their emotions and stresses during appointments. These fathers highlighted the need for a “check in and touch base for dads” (Thomas, Father, early 30s) with professionals and emphasised that it was important for midwives to talk to fathers about their emotional challenges they experience during the early stages of fatherhood, “even a simple act of just asking, so how are you finding it? How are you feeling? Makes you feel a lot more supported” (Michael, Father, early 30s).

Few of the fathers expressed that their well-being was considered, and that additional emotional and mental health support was provided if needed when disclosing unique cases such as their baby was kept in an Neonatal Intensive Care Unit (NICU), “throughout we’ve been able to access support through a local charity whilst within the ward” (Paul, Father, late 30s) and “they had peer support things in there for dads. But then once you come out of that environment. Then that kind of disappears” (Dale, Father, early 40s). However, fathers argued that all fathers’ emotional well-being should be considered throughout, and accessible support should not be limited to when in the NICU. As Dale highlights through the use of ‘disappears’*,* the expectations are for fathers to carry on supporting the family independently after potential trauma. These differing experiences recognise the benefit of inclusive emotional support throughout the perinatal period. Practitioners supported the importance of being aware of fathers’ emotional state and simply asking questions, including “what are you most worried about and what is that like?” (Avery, SCS, Social Worker). They agreed that a healthy well-being, including both parents, is essential to support the child. Although both fathers and professionals emphasised the importance of prioritising mothers’ well-being during the perinatal period, the findings suggest that traditional masculine expectations and norms of emotional stoicism may also contribute to the limited recognition of fathers’ own emotional support needs. As illustrated by Dale’s account, fathers often described prioritising the needs of their partner and family while minimising or suppressing their own emotional experiences.

Most fathers and practitioners therefore highlighted the need for an alternative direction within healthcare and social care; a “whole family support approach” (Phoebe, VCSE, Operational Employee) and *“*inclusivity of parents as a whole” (Ross, Father, late 30s) were considered to benefit and support the whole family directly to influence positive family and childhood development.

Based on work experiences, practitioners felt that the importance of a healthy relationship between parents was undervalued; they felt a healthy relationship was paramount for a healthy parenting transition. This included providing relationship support on how to support each other and correspondence with both parents. Similarly, this was also discussed by many fathers in relationships with the mother of their child, with a healthy relationship approach being prioritised. They also highlighted the need for support towards each other, especially from their partners, when fathers often expressed limited support networks. These fathers stated that support should be relayed to both parents to encourage engagement from fathers, in time building communication and a positive relationship with their partner.

“How many times you see a baby come along, and not long later a relationship breaks down. What I imagine, is that there’s a huge aspect to that with regards to the preparation and the inclusivity of parents, as opposed to focusing on one of them.” (Ross, Father, late 30s)

#### Subtheme 2: “What is antenatal information?” Strive for inclusion and equity of antenatal resources

Fathers, biological and adoptive, reflected on the need for inclusion and equity of resources and information as preparation. Fathers often reported that they were not given antenatal information: “What is antenatal information? I didn’t get any.” (Liam, Father, early 30s). It was stated by fathers that quite often parenting, or antenatal information, was relayed to them through either their partner or accessing online: “I guess I Googled everything; it was the only experience I had” (Kwame, Father, late 20s). Relying primarily on in-depth education or information from their partner was seen as unhelpful and unsupportive when, at times, their partner struggled to contain and communicate it alone. This was recognised within certain fathers who were in full-time employment or working multiple part-time jobs due to low socioeconomic status, who were sometimes unable to attend perinatal appointments, due to work and financial commitments in comparison to other fathers, who were, for example, full-time students who could “I went to all the scans and appointments, which, I think, is unusual” (Father, Michael, early 30s).

However, overall, many of the fathers who did attend appointments expressed a lack of resources and information communicated to them: “for my wife it was a lot (information/resources) would have been more beneficial for me to have” (Finn, Father, mid 20s). Similarly, this was also shared by practitioners, whereby they felt fathers should also be entitled to correct information and education from professionals and within resources: “I don’t think that dads are given much information at all other than what a partner tells them.” (Claire, SCS, Senior Project Manager).

As mentioned in theme 1, fathers felt inspired to learn and to fulfil their role as a supportive father figure during pregnancy through preparation. These fathers expressed, however, that they did not want information and resources created specifically for themselves; fathers emphasised the preference for information provided similarly to mothers, with resources covering mother and father queries and needs to efficiently support their partner. They stated that it is important that this information is signposted to both parties inclusively in apps/resources and healthcare settings, as it is possible that this would help both parents’ transition to parenthood more positively. Accounts from both fathers and professionals suggested that the increasing expectation for fathers to adopt more emotionally engaged and supportive roles during pregnancy and early parenthood may be constrained by enduring and underlying gendered assumptions within perinatal care. Specifically, participants described how traditional approaches that position mothers as the primary recipients of perinatal information and support may inadvertently limit the recognition of fathers’ own informational and emotional support needs. These practices may contribute to the continued underdevelopment of father-inclusive resources and education within perinatal services, limiting opportunities to support the whole family.

“I just think more information for the duo the parents, as opposed to a new mother it should be more of an include.” (Ross, Father, late 30s)

It was recognised by practitioners that provision of this antenatal support would allow fathers to feel validated by practitioners within their experiences, for example, providing crying support and feeding skills to reassure them, “it’s normal, don’t worry it doesn’t mean they don’t love you when they cry!” (Jess, SCS Project Development Officer). This support was recognised as equipping fathers “on how to adapt” (Thomas, Father, early 30s) and a “simple way to improve mental health” (Will, VCSE, Director of Organisation). Fathers expressed a preference to acquire information and skills from resources more logically to rationalise any anxiety or mental distress: “all the things that could happen or might not happen, or things that could go wrong and like that would be really good.” (Noah, Father, early 30s). These fathers, who were in relationships with the mother of their child, often shared experiences of feeling at a loss in how to support their partner with the challenges of parenting, due to not receiving information.

Adoptive fathers also acknowledged issues and a loss regarding antenatal education. They valued the need for parenting support and fatherhood resources, due to the absence of antenatal and pregnancy appointments entirely: “maybe prenatal pre-birth classes, you know, if you're a mum with a baby, you go to kind of like scans and all of that sort of stuff. So maybe information for me and my partner” (Alfie, Father, early 30s). They particularly highlighted the need for “a big section on mental health and well-being within antenatal classes” (Alfie, Father, early 30s) as a preventative approach to inform them about potential emotional or mental health implications/challenges as a response to lifestyle adjustments when becoming a father. Many challenges were experienced by all fathers; however, adoptive fathers, such as Alfie, expressed that adoption can be very unpredictable, for example, adopting two children as opposed to one, of differing ages, with often underlying experiences of childhood trauma.

#### Subtheme 3: “A system furnished for mum” development of service infrastructure and policies targeting maternal caregiver bias

Fathers in this study recognised and supported the need for perinatal services to prioritise the clinical care of mothers. However, they also identified appropriate opportunities within existing care pathways for greater recognition of inclusion, and support for fathers’ own health and well-being during the perinatal period. Both fathers and professional participants stated that there is a need for development promoting physical inclusion and fatherhood engagement within current health and social care services. Although few fathers did disclose their positive experiences and inclusion from professionals within these services, stating a non-isolating transition, “I never felt like a second-class citizen in there” (Conor, Father, mid 30s). Many other fathers expressed contradictory experiences; they desired to be considered an intrinsic part of the perinatal journey; however, they often expressed feeling invisible:

*“*I can say for me it wasn’t, but towards my wife it was like a lot like the times we were there like they will address her a lot, for me l like I feel that I wasn’t” (made to feel an intrinsic part of the perinatal journey).*”* (Finn, Father, mid 20s)

Physical, environmental and cultural barriers deterred these fathers from getting involved. This included bright, feminine colour landscapes and uninviting aesthetics within services, the use of maternal terminology “they were kind of like mother and baby centres” (Phoebe, VCSE Operational Employee) and institutional prejudice of female-dominant workforces that lacked a fatherhood perspective. When interpreting these accounts, it is possible that perinatal healthcare services continue to operate within gendered assumptions that position mothers as the primary recipients of care and support. Participants described how maternal-focused environments, language and service practices may inadvertently limit recognition of fathers’ roles and needs, contributing to experiences of fatherhood invisibility within the perinatal period.

Both fathers and practitioners recognised that healthcare and social care services infrastructure and systems were designed to focus on mothers as a primary caregiver insofar as Information Technology (IT) systems were reportedly set up with the mother as a default contact for correspondence. As stated by Simon, “they’ve kind of designed dads out of the system; they don’t exist because of the way that IT systems are built.” (Simon, VCSE, Director of organisation). One participant also pointed out how letters were addressed to mothers only, excluding fathers and partners, especially when communication and contact were initiated about the child. These systems were seen to frustrate these fathers as well as contribute to fathers’ feeling unworthy of their parental role, influencing their mental health negatively. Participants’ accounts suggest that organisational systems within perinatal services may be shaped by institutional cultures that position mothers as the primary recipients of care. These structures may unintentionally limit opportunities for father inclusion through routine practices, such as communication pathways, information provision and service engagement processes.

One practitioner pointed out, “there should be the expectation that dads attend appointments” (Claire, SCS, Senior Project Manager). This quote emphasises that it is not solely system exclusion that needs to change to make this more feasible for fathers, but professionals’ acceptance of father involvement within healthcare and social care services as an ‘expectation’. Many of the practitioners interviewed acknowledged that there was a need for themselves to take responsibility and initiate inclusion, providing fathers with their *“*own choice” (Jess, SCS, Project Manager) to be informed and aware. This also included influencing positive engagement within appointments and breastfeeding groups, as opposed to fuelling continuous rejection and supporting maternal caregiver bias constructs.

Most fathers expressed negativity around the limited allocated paternity leave within the UK, with one father referring to it as *“*terrible in this country (UK)” (Noah, Father, early 30s) in comparison to other countries, such as Scandinavian countries. Many of the fathers felt frustrated and cheated due to finding it was much harder to develop a bond with their child, with limited time in comparison to their partner. This often had a detrimental impact on their father-child relationship postnatally and indirectly the fathers’ well-being: “the bond that she created in those nine months postpartum it’s like obviously instrumental to the relationship with him now.” (Noah, Father, early 30s). One father, Michael, however, discussed a more positive paternity leave and postnatal experience, due to the advantage of no restrictions on how much time he could take off due to his current student status. Having the additional time off meant he was able to attend support groups with his partner, exchanging feelings and thoughts with others, as well as spending time with and supporting his family, resulting in a more mental and physical supportive experience “getting that amount of time to spend with your child……and a really nice period because you can just talk about everyone’s experiences and go to NCT classes” (Michael, Father, early 30s).

Fathers, particularly those who identified as white British within the UK, reflected that they felt stuck in a generational gap of adopting more modern approaches, yet without the broader societal changes and actions required within law, policies and institutional systems to support the inclusion and rights of fathers. It was recognised by all the participants the prioritisation of clinical care of mothers; however, by adopting a maternal caregiver bias within systems and policies was acknowledged as outdated, leading to unattainable expectations. The findings suggest that enduring societal expectations surrounding paternal roles, alongside wider uncertainty about contemporary fatherhood identities, may contribute to the gendered structures underpinning perinatal systems, practices and policies. These influences may shape how fathers are recognised and included within perinatal care, contributing to experiences of marginalisation and limited support. Indeed, practitioners highlighted that “there needs to be a huge cultural shift in health services” (Claire, SCS, Senior Project Manager) and that from a political stance “politicians need to understand the uniqueness of dads, and the benefits to babies to commission engagement” (Nathan, VCSE, Operational Manager).

## Discussion

The aim of this study was to explore fathers’ mental health and well-being experiences and their support needs within the perinatal period, as well as current viewpoints of perinatal services from the perspective of fathers and professionals. Two overarching themes were identified within the reflexive thematic analysis: (1) ‘The Pregnant and Postnatal Man’ and (2) Removing the cloak of fatherhood invisibility. Consistently presented throughout this study’s findings and across current literature are the substantial transitional and psychological challenges that many biological and adoptive fathers can face within the perinatal period. For example, stress, anxiety, low mood, confrontation, social isolation, self-doubt, exhaustion and financial pressures. Previous systematic reviews have highlighted increased paternal stress within the perinatal period, leading to mental health problems such as perinatal depression and anxiety.^42–45^ Previous research recognises that fathers within contemporary parenting approaches are expected to combine a multitude of roles, including breadwinning and providing emotional and practical care to their children.^17 18 28^ As shown within this research, expectations of fathers, particularly from majority ethnicities within the UK, balancing multiple roles (employee, father and husband) with limited support within the perinatal period have impacted their mental and physical capacity and health. This has led to coping mechanisms that were considered unhelpful by mental health professionals, including work escapism and avoidance of the family environment, contributing towards a cumulative decline in their mental health.

Within this research, both fathers and professionals placed importance on the role of services and healthcare, offering a ‘whole family approach’ to inclusivity and support within the perinatal period for mothers, fathers and partners. Findings expressed possibilities of how to adopt a more father-inclusive environment within healthcare services, maternity services and social care, as well as across policies to support the well-being of the whole family. Previous research has highlighted that fathers may experience feelings of invisibility and uncertainty when engaging with maternity services, which have been associated with maternal-focused service structures, communication practices and experiences of limited inclusion.^46 47^ While the prioritisation of women’s health and safety remains central to maternity care, services organised around the pregnant woman as the primary recipient of care may unintentionally limit recognition of fathers’ roles and support needs. Furthermore, health professionals may encounter organisational and structural barriers to engaging fathers, including established institutional cultures and gendered assumptions surrounding caregiving and parental roles.^12 48^ Research has acknowledged the need for increased recognition of fathers’ involvement.^48^ Recognition of fathers has been reported to increase confidence in parental responsibilities, contributing to positive psychological and social outcomes for the father and family.^49^ Within this study, fathers and professionals acknowledged aspects of exclusion directed to fathers, including physical and environmental barriers, use of maternal terminology, institutional prejudice and lack of fatherhood perspective. Participants also shared similar frustrations and discrimination from potential maternal caregiver bias within policies, including paternity leave. Ndzi and Holmes^50^ state that paternal perinatal mental health can be impacted when paternity leave entitlement is too short, not allowing fathers to adjust to the fatherhood transition. Similarly, Hobbs^51^ recognised that extended paternity leave would promote fathers’ psychological well-being by allowing time to support the family’s well-being and self-reflect, contributing towards positive paternal perinatal mental health.

Within this study, all fathers were in relationships with the mother of their child, yet they recognised less of a ‘family approach’ when it came to emotional support within the perinatal period. Past research and guidelines support a more women-centred approach regarding support, with most maternity and child-health services designed to rightly so prioritise the needs of the mother and child.^52 53^ However, assessment of fathers’ well-being and mental health is not routinely included.^53^ Within this study, these fathers respected their partners’ needs as a priority; however, they felt that they were not recognised within services as requiring emotional support, even when meeting expectations of supporting their partner and family. As identified by Steen and Downe,^49^ fathers are less able to support their partner and present a positive parenting experience without father support and inclusion. This was emphasised by fathers within this research whose partners were diagnosed with perinatal mental health disorders; fathers desired mental and emotional check-ins within appointments. Recent research by Hodgson and Jenkin^13^ also supports regular check-ins with fathers within appointments or a separate phone call after. There are limited guidelines or funds targeted to the needs of fathers within maternity services that can be used by health professionals.^53^ Some clinicians may also not be entirely comfortable engaging with fathers and responding to their well-being challenges due to a lack of training and confidence.^54^ When concerning fatherhood well-being and mental health challenges/concerns, there is a systemic bias within clinical training and practise.^55^

Additionally, fathers and professionals within this research highlighted a need for equity and inclusion within antenatal resources, with information focusing on both mother and father queries and needs to support their partner and family well-being. Antenatal education would also benefit from addressing fathers’ needs.^56^ Fathers valued information being designed and presented inclusively in healthcare settings (appointments and apps). Many barriers and norms can influence poor resource allocation and customs of antenatal education.^57^ According to Charlton,^58^ it is important that professionals prioritise the provision of information at the beginning of the process and throughout. Mental health and emotional well-being classes were emphasised by fathers, particularly adoptive fathers, to inform them on potential emotional and mental health implications. Limited information about mental health issues or conditions has been recognised by fathers within antenatal classes.^13^ Hodgson and Jenkin^13^ similarly highlighted the need for equivalent information and support regarding the mental well-being of the whole family.

### Strengths and limitations

The qualitative approach of father and professional exploration provided new insight and a richer understanding of fathers’ mental health and well-being experiences, their support needs and current viewpoints and perspectives of perinatal services. Professional perspectives are especially limited within the literature exploring this topic. This study identified contrasting and parallel viewpoints on the support needs of fathers specific to this study, adding understanding of any additional contextual factors associated with perinatal services for effective and feasible policy and practice recommendations. Multiple recruitment strategies were employed within the sampling approach, resulting in heterogeneity of participants. Fathers of differing backgrounds, ages and ethnicities, living across different places within the NENC, were recruited. Professionals were also recruited from various organisations/charities and differing roles within children’s social care services.

Although a primarily broad and diverse sample was obtained from biological fathers, recruiting adoptive father participants proved to be more challenging; therefore, interpretations and conclusions made within the analysis were restricted. An additional limitation of the sample was that all fathers who participated were in a relationship or living with the mother of their child/children. The composition of the study sample limits the transferability of the findings to fathers whose experiences are currently under-represented, including single, adoptive and ethnically diverse fathers, as well as fathers who do not speak English. The findings may also be influenced by the predominance of participants who were in ongoing intimate relationships, meaning that the experiences and support needs of fathers with different family structures or social circumstances may not be fully reflected. Future research should explore the experiences of adoptive fathers, single and widowed fathers, fathers in same-sex relationships, and fathers from ethnically diverse and non-English-speaking communities. Examining how cultural, social and family contexts shape fathers’ experiences of perinatal support would strengthen the evidence base and inform the development of more inclusive and equitable father-inclusive perinatal services.

Many of the professional participants from the VCSE sector had previously worked within the NHS or healthcare settings, with a few currently working in the NHS, therefore referring to and reflecting at times on these work experiences. However, although intended and attempted, no explicit health professionals were recruited directly through the NHS for this study; obtaining this sample proved to be difficult and was not within this research’s ambit. A further limitation is the absence of frontline NHS clinical and managerial perspectives. As many of the recommendations relate to the organisation and delivery of maternity services, the interpretation of findings relating to healthcare service design should be considered in the context of this omission. Although the study provides valuable insights into fathers’ experiences and perspectives from VCSE and social care organisations, the recommendations would benefit from further exploration and validation with NHS clinicians, managers and service leaders to ensure they are feasible, contextually appropriate and applicable within routine maternity care. Additional views from healthcare professionals would have allowed more specific and relatable experiences and examples to fathers within maternity and healthcare services, contributing to more relevant practice and policy implications and recommendations on support. Table 4 presents the practice, policy and research implications associated with this study.

**Table 4:** Practice, policy and research implications

Practice:	The findings of this study suggest that there is a need for a ‘whole family’ inclusive support approach within perinatal services. It is recommended that health systems alter their perceptions and infrastructures to be more inclusive of fathers’ needs and eliminate systemic inequalities. Healthcare providers in maternity/perinatal services need sufficient time to address fathers’ well-being and mental health within appointments and to signpost. There is a need for the adoption of gender inclusive mental health/emotional training, as well as updated National Institute for Health and Care Excellence (NICE) clinical guidelines to support clinicians when supporting fathers’ needs.^53 59^
Policy:	Paternity leave within the UK was recognised as unsatisfactory by fathers, with most of these fathers taking the maximum statutory 2 weeks paternity leave and not shared parental leave (SPL). Low uptake of the SPL policy over the years has been recognised due to a lack of awareness and the existence of the legislation, discrimination in the workplace and non-extension of pay to SPL.^50 60 61^ Uptake is slowly improving; further evaluation (survey/qualitative) of the SPL policy, assessing variables influencing uptake and how to improve accessibility, is important, considering the mental health benefits of extended paternity leave.^51^ It is recommended that healthcare policy makers and commissioners should recognise fathers’ input and seek to address fathers’ mental health and well-being, to support future service provision and commission engagement of fathers within the perinatal period.^59^
Research:	Further qualitative exploration on the support needs of socially and economically diverse fathers, considering the strong correlations between social determinants and poor mental health.^62^ Exploration of adoptive fathers as part of same sex couples’ experiences is required, as the existing evidence is limited. Additional exploration of younger fathers (up to 25 years of age) is required to understand their unique experiences and any associations with the education system in the UK. Exploration of single or widowed fathers’ perinatal journeys should be prioritised to address alternative narratives and support needs. Qualitative exploration with health professionals (midwives, practitioners and nurses) within perinatal services to inform system and service development.

## Conclusions

This research has highlighted important impacts from both the public and professionals that may relate to practice (perinatal services) within the UK. The findings suggest that greater father inclusion within perinatal services, policies and antenatal education may help fathers feel more recognised and supported within the perinatal journey. Recognition of fathers’ emotional well-being and mental health, by adopting a ‘whole family’ emotional support approach within perinatal services, was considered important for fathers so that they can support the family dynamic, influencing a positive transition to parenthood for the whole family.

## Supplementary material

10.1136/bmjopen-2026-121953 online supplemental file 1

## Data Availability

All data relevant to the study are included in the article or uploaded as supplementary information.
