# Supplementary material for: Understanding fathers’ perinatal mental health and well-being support needs: a qualitative study of fathers’ and professionals’ perspectives in North East England and North Cumbria, UK
Source: BMJ Open. 2026 Sep 4;16(9):e121953. doi: 10.1136/bmjopen-2026-121953 (PMC13548270; doi:10.1136/bmjopen-2026-121953)
Supplement: Supplementary file 1 [file bmjopen-2026-121953.reviewer_comments.pdf]

PEER REVIEW HISTORY

BMJ Open publishes all reviews undertaken for accepted manuscripts. Reviewers are asked to complete a checklist review form and are provided with free text boxes to elaborate on their assessment. These free text comments are reproduced below.

ARTICLE DETAILS

Title (Provisional)

Understanding fathers' perinatal mental health and well-being support needs: a qualitative study of fathers' and professionals' perspectives in North East England and North Cumbria, UK

Authors

Watkins, Ashleigh Elizabeth; El Zerbi, Catherine; McGovern , Ruth; Rankin, Judith

---

VERSION 1 - REVIEW

**Reviewer**	**1**
**Name**	**Tarrant, Anna**
**Affiliation**	**Lincoln University , School of Social and Political Science**
**Date**	**29-May-2026**
**COI**	**None**

This is an interesting empirical article reporting on the perspectives of fathers and professionals associated with perinatal support on the how fathers experience isolation, exclusion and limited support in perinatal contexts. The empirical inclusion of both fathers and professionals, particularly from VCSE and social care sectors, is a notable strength and provides important insights into how paternal support needs are experienced and perceived across service contexts in the peri-natal period. The findings are highly relevant to contemporary debates surrounding father inclusion, perinatal mental health, and family-centred approaches to care, highlighting fathers' experiences of emotional isolation, limited recognition within perinatal services, and perceived exclusion from antenatal education and support. The study also offers useful practice and policy implications regarding the need for more inclusive, family-focused approaches within perinatal services.

However, there are several areas where the manuscript could be strengthened. The findings provide convincing evidence that many fathers experience exclusion and marginalisation within perinatal services, supporting the authors' notion of a 'cloak of fatherhood invisibility'. However, the analysis could be strengthened by further unpacking the mechanisms through which this invisibility is produced and maintained. At present, the theme is well evidenced descriptively but would benefit from deeper analytical engagement

with the institutional, cultural and structural factors underpinning fathers' experiences of invisibility. This focus would then also strengthen the alignment between critical realism as the philosophical orientation and the analytical interpretation presented in the findings and discussion.

The discussion of 'maternal caregiver bias' would benefit from greater critique. While participants clearly described experiences of exclusion within services, the manuscript should more explicitly distinguish between the often necessary clinical prioritisation of mothers within maternity care and the unintended marginalisation of fathers. Such clarification would help to circumnavigate interpretations that position father inclusion and women-centred care as competing priorities.

A further limitation concerns the professional sample. Despite the study's focus on healthcare, maternity, and perinatal services, no NHS professionals were recruited. This limitation is acknowledged but warrants greater emphasis given that many of the recommendations are directed towards healthcare systems and maternity services. Additional discussion regarding how the absence of NHS voices may have influenced the findings would strengthen the paper.

More generally, the manuscript would also benefit from some refinement in reporting.

At times, claims are made that extend beyond the scope of the sample. The findings certainly provide important insights into participants' experiences; however, greater caution may be warranted when making broader claims about fathers' needs and experiences given the specific characteristics and limitations of the sample, especially in the conclusion. By way of example, it is claimed that 'Fatherhood inclusion and equality within perinatal services, policies, and antenatal resources/education are essential for fathers to be considered intrinsic and supported within the perinatal journey.' This might be more effectively framed as: 'The findings suggest that greater father inclusion within perinatal services, policies and antenatal education may help fathers feel more recognised and supported within the perinatal journey'.

Relatedly, although the study includes participants from different ethnic backgrounds and family circumstances, this sample remains relatively small, all participating fathers were reportedly in relationships, and recruitment of adoptive fathers and fathers from more disadvantaged circumstances was limited. Greater caution is therefore required when discussing the needs and experiences of 'fathers' more broadly.

There are some minor grammatical and stylistic issues throughout the manuscript that would benefit from careful proofreading. In addition, the rationale for claiming saturation should be avoided, particularly given the qualitative nature of the study and ongoing debates regarding the compatibility of saturation with reflexive thematic analysis.

I'd suggest avoiding the use of collective 'we' in the background section by being more specific about wider global and generational trends in involved fatherhood. Sociological research might be cited here.

The background section overall would benefit from with a stronger, more cohesive argument. There are lots of claims to a lack of research, where the arguments could be more productively focused on what we do know from existing literature to really situate the findings.

On page 4, the authors refer to the sidelining of fathers in services without using this language/concept. The work of Neale and Tarrant (2024) and Tarrant (2025) might usefully be cited here to support theorisation of this sidelining. The broader literature also refers to father-inclusion rather than fatherhood-inclusion, so this language might better reflect the wider literature.

Overall, this is a timely and important study that makes a valuable contribution to the literature on paternal perinatal mental health and father-inclusive practice. The findings have clear implications for policy and service development and subject to revisions, the manuscript has strong potential.

**Reviewer**	**2**
**Name**	**Gu, Chunyi**
**Affiliation**	**Obstetrics and Gynecology Hospital of Fudan University**
**Date**	**30-May-2026**
**COI**	**None**

Thank you for giving me this opportunity to review this manuscript. This qualitative study aims to explore fathers' mental health and well-being experiences, support needs within the perinatal period, and current viewpoints of perinatal services (maternity, healthcare and social care services) from both fathers' and professionals' perspectives. This topic is timely and relevant. And the manuscript is generally well-written and structured. However, several methodological and analytical limitations need to be addressed before publication.

1. Introduction: The authors state that "Prior research has identified that fathers are up to 47 times more likely to be 100 identified as a suicide risk in the perinatal period than at any other point in their lives, highlighting the importance of exploration around fathers' mental health in the perinatal period, specifically within the NENC (19). Here the reference (19) (Quevedo et al., 2011) is outdated. Please add a brief qualifier or consider citing a more recent literature.

2.Methods-Setting: The description of the "Setting" subsection is currently too brief. The authors state only that fathers resided in the NENC and that professionals were recruited from VCSE and social care services. No contextual information is provided, which limits the

reader's ability to assess the transferability of the findings to other regions or populations. Providing more details will enhance the transparency and reproducibility of the study.

3. The authors state in the limitations that all fathers who participated were in a relationship with or living with the mother of their child. However, this crucial sample characteristic is not reported in the Results section. Please add relationship status (e.g., "living with partner/mother of child") to Table 2 or include it explicitly in the narrative description of the father sample.

4. As the father sample is predominantly White British (12/21) (Results section- Table 2), and all fathers were in a relationship with the mother of their child, this limits the transferability of findings to single fathers, fathers from minority ethnic backgrounds, et al. While the authors acknowledge this limitation, the discussion should more explicitly state how these subgroups' support needs may systematically differ. Future research priorities should be clearly identified.

5.Inconsistency between inclusion criteria and actual sample： Table 1 lists "NHS professional" as an inclusion criterion for professionals. However, the authors later acknowledge that no health professionals were recruited directly through the NHS. This discrepancy should be clarified. Either the inclusion criteria should be revised to reflect the actual sample (VCSE and social care only), or the authors should explain why no NHS professionals participated and how this affects the interpretation of findings related to healthcare service design.

VERSION 1 - AUTHOR RESPONSE

Reviewer Comments	Response to reviewer comments	Location in manuscript
**Reviewer 1 Comments:**<br><br>1. However, there are several areas where the manuscript could be strengthened. The findings provide convincing evidence that many fathers experience exclusion and marginalisation within perinatal services, supporting the authors' notion of a 'cloak of fatherhood invisibility'. However, the analysis could be strengthened by further unpacking the mechanisms through which this invisibility is produced and maintained. At present, the theme is well evidenced descriptively but would benefit from deeper analytical engagement with the institutional, cultural and	Thank you for all of your comments and feedback. I have taken it all on board and made amendments to the manuscript.<br><br>Thank you for this helpful feedback. In response, I have strengthened the **'Removing the Cloak of Fatherhood Invisibility'** theme by further developing the analysis to identify and explain the mechanisms through which fathers' invisibility is produced and sustained within the perinatal period.<br><br>Specifically, I have expanded the discussion of how traditional	Theme 2 (all sub-themes) (line 400 – 606)

structural factors underpinning fathers' experiences of invisibility. This focus would then also strengthen the alignment between critical realism as the philosophical orientation and the analytical interpretation presented in the findings and discussion.	masculine norms, including expectations of emotional stoicism and self-reliance, limit recognition of fathers' emotional needs and reduce opportunities for support. The analysis also examines how these gendered expectations can constrain expressions of affectionate fatherhood and involvement, while contributing to the limited provision of father-inclusive information and education.<br><br>In addition, I have developed the analysis of healthcare systems and organisational practices, demonstrating how services frequently operate within gendered assumptions that position mothers as the primary recipients of perinatal care. This is reflected in maternal-focused environments, language and communication practices, which may inadvertently contribute to fathers' experiences of exclusion. I have also strengthened the discussion of institutional mechanisms, including service infrastructure and routine practices (e.g. failing to record fathers' contact details or communicate directly with them), to illustrate how organisational processes can reinforce father invisibility.<br><br>Finally, I have extended the discussion to consider how these institutional practices are shaped by broader societal and cultural expectations surrounding fatherhood, including traditional gender roles, uncertainty around contemporary father identities and policy contexts such as parental leave. Together, these revisions provide a more explicit account of the interconnected social, cultural and organisational mechanisms underpinning fathers' experiences of invisibility during the perinatal period.	

2. The discussion of 'maternal caregiver bias' would benefit from greater critique. While participants clearly described experiences of exclusion within services, the manuscript should more explicitly distinguish between the often-necessary clinical prioritisation of mothers within maternity care and the unintended marginalisation of fathers. Such clarification would help to circumnavigate interpretations that position father inclusion and women-centred care as competing priorities.	Thank you for this helpful comment.<br><br>In response, I have revised the manuscript to distinguish more clearly between situations where the prioritisation of mothers reflects an appropriate clinical focus and those where fathers' exclusion appears to result from unintended organisational or policy-related practices. Throughout the manuscript, I have been more explicit when interpreting fathers' accounts to ensure this distinction is consistently maintained.<br><br>These revisions are intended to avoid framing father inclusion and woman-centred maternity care as competing priorities. Instead, the manuscript now emphasises that improving father inclusion can be achieved alongside, and in ways that support, the continued delivery of high-quality, woman-centred care.	Throughout theme 2 (sub-theme 3) and within the discussion (Line 631-638)
3. A further limitation concerns the professional sample. Despite the study's focus on healthcare, maternity, and perinatal services, no NHS professionals were recruited. This limitation is acknowledged but warrants greater emphasis given that many of the recommendations are directed towards healthcare systems and maternity services. Additional discussion regarding how the absence of NHS voices may have influenced the findings would strengthen the paper.	Thank you for this helpful feedback.<br><br>In response, I have expanded the discussion to more explicitly acknowledge how the absence of NHS staff perspectives may have influenced the interpretation of the findings and the recommendations arising from this study. Given that many of the recommendations relate to the organisation and delivery of maternity services, the lack of frontline clinical and organisational NHS perspectives limits the extent to which the findings can fully account for the practical, operational and resource-related considerations involved in implementing service change.<br><br>I have therefore clarified that the implications for practice should be interpreted with this limitation in mind. While the findings provide	Discussion (Above practice and policy and research implications) (Line 713-719)

	valuable insights into fathers' experiences and identify opportunities for improving father inclusion, the recommendations would benefit from further exploration and validation with NHS clinicians, managers and service leaders to ensure they are feasible, acceptable and applicable within routine maternity care. Overall, I have identified this as a limitation of the present study and a priority for future research.	
4. At times, claims are made that extend beyond the scope of the sample. The findings certainly provide important insights into participants' experiences; however, greater caution may be warranted when making broader claims about fathers' needs and experiences given the specific characteristics and limitations of the sample, especially in the conclusion. By way of example, it is claimed that 'Fatherhood inclusion and equality within perinatal services, policies, and antenatal resources/education are essential for fathers to be considered intrinsic and supported within the perinatal journey.' This might be more effectively framed as: 'The findings suggest that greater father inclusion within perinatal services, policies and antenatal education may help fathers feel more recognised and supported within the perinatal journey'.	Thank you for this helpful comment.<br><br>I agree that some statements were framed too broadly and extended beyond what could be supported by the study sample. In response, I have revised the manuscript throughout to ensure that the language more accurately reflects the scope of the data and avoids making generalised claims about fathers' experiences.<br><br>In particular, I have amended the Abstract and Conclusion to ensure that the findings are presented as relating to the experiences and perspectives of participants in this study, rather than to fathers more broadly. These revisions better align the interpretation and conclusions with the study design and the evidence presented.	Throughout findings and discussion sections.<br><br>Abstract (Line 56-57)<br><br>Conclusion (Line 729-733)
5. Relatedly, although the study includes participants from different ethnic backgrounds and family circumstances, this sample remains relatively small, all participating fathers were reportedly in relationships, and recruitment of adoptive fathers and fathers from more disadvantaged circumstances was limited. Greater caution is therefore required when discussing the needs and experiences of 'fathers' more broadly.	Thank you for this helpful feedback. In response to this and the preceding comment, I have revised the manuscript, particularly the Findings and Discussion sections, to ensure that interpretations are consistently situated within the characteristics of the study sample. Where fathers' experiences and needs are discussed, I have provided additional contextual detail regarding participants' backgrounds, including their	Throughout findings and discussion sections.

	relationship to the child's mother and relevant sociodemographic characteristics, where appropriate.<br><br>I have also revised the wording throughout to ensure that the findings and implications are explicitly presented as relating to the fathers who participated in this study, rather than implying broader generalisability. These changes better align the interpretation of the findings with the study sample and qualitative design.	
6. There are some minor grammatical and stylistic issues throughout the manuscript that would benefit from careful proofreading. In addition, the rationale for claiming saturation should be avoided, particularly given the qualitative nature of the study and ongoing debates regarding the compatibility of saturation with reflexive thematic analysis.	Thank you for this feedback, I have proof read the manuscript and made amendments to grammatical errors, as well as reworded/added words to sentences due to some stylistic issues.<br><br>I have also removed the rationale for claiming saturation due to the qualitative nature of the study and especially due to the compatibility of saturation with reflexive thematic analysis.	Throughout the manuscript<br><br><br>Data collection section (Line 234)
7. I'd suggest avoiding the use of collective 'we' in the background section by being more specific about wider global and generational trends in involved fatherhood	Thank you for this comment, I agree to not use the use of collective 'we' and have removed it and replaced the sentence focusing on the wider generational shift of the fatherhood identity and social expectations, as well as touching on fatherhood involvement in the past 20 years and moving away from traditional approaches due to global trends also influencing change.	Background section (Line 78-86)
8. The background section overall would benefit from with a stronger, more cohesive argument. There are lots of claims to a lack of research, where the arguments could be more productively focused on what we do know from existing literature to really situate the findings.	Thank you for this helpful feedback.<br><br>In response, I have revised the Background to place greater emphasis on the existing evidence base rather than focusing primarily on gaps in the literature. Specifically, I have incorporated additional relevant research to provide a more balanced overview of what is currently known about	Background section (Line 91-140)

	fathers' perinatal experiences and support needs.<br><br>I have also revised the rationale for the study to demonstrate more clearly how the current findings build on and complement the existing literature, while identifying the specific areas where further evidence is needed. These revisions provide a stronger justification for the study and better situate its contribution within the wider evidence base.	
9. On page 4, the authors refer to the sidelining of fathers in services without using this language/concept. The work of Neale and Tarrant (2024) and Tarrant (2025) might usefully be cited here to support theorisation of this sidelining. The broader literature also refers to father-inclusion rather than fatherhood-inclusion, so this language might better reflect the wider literature.	Thank you for this helpful suggestion and for directing me to this additional literature. I have revised the manuscript to incorporate the recommended evidence, which strengthens the theoretical framing of fathers' exclusion and better situates the findings within the wider literature on father inclusion.<br><br>Specifically, I have drawn on Tarrant's work to expand the discussion of how institutional structures, professional practices and gendered assumptions can contribute to the marginalisation of fathers as caregivers within maternity services. I have also incorporated literature highlighting the growing recognition of fathers' involvement in the perinatal period, while acknowledging the organisational and systemic barriers that can limit health professionals' ability to engage fathers effectively. These additions provide a stronger theoretical foundation for interpreting the study findings and place them within the broader evidence base on father-inclusive maternity care.	Background section (Line 91-107)
**Reviewer 2 Comments:**<br><br>1. Introduction: The authors state that "Prior research has identified that fathers are up to 47 times more likely to be 100 identified	Thank you for this helpful feedback.	Background section (Line 114-116)

as a suicide risk in the perinatal period than at any other point in their lives, highlighting the importance of exploration around fathers' mental health in the perinatal period, specifically within the NENC (19). Here the reference (19) (Quevedo et al., 2011) is outdated. Please add a brief qualifier or consider citing a more recent literature.	In response, I have updated the manuscript to include a more recent reference supporting this statement. Specifically, I have cited findings from the **Paternal Suicide in the 1001 Critical Days** study (2025), which reports that an estimated two to three infants lose their fathers to suicide each week in the UK. This revision ensures that the statement is supported by the most up-to-date available evidence.	
2. Methods-Setting: The description of the "Setting" subsection is currently too brief. The authors state only that fathers resided in the NENC and that professionals were recruited from VCSE and social care services. No contextual information is provided, which limits the reader's ability to assess the transferability of the findings to other regions or populations. Providing more details will enhance the transparency and reproducibility of the study.	Thank you for this helpful comment.<br><br>In response, I have expanded the manuscript to provide greater contextual detail about the study setting and population. Specifically, I have clarified that the study was conducted at Newcastle University, Newcastle upon Tyne, UK, as part of a funded PhD within the NIHR Applied Research Collaboration North East and North Cumbria (ARC NENC), and that all data were collected online.<br><br>I have also strengthened the Methods/Setting section by providing additional contextual information about the North East and North Cumbria region, including its comparatively high levels of socioeconomic deprivation and suicide, to help situate the study findings within the wider regional context. In addition, I have noted the absence of regional data on paternal perinatal mental health, highlighting an important evidence gap that further supports the rationale for the study. These revisions provide readers with a clearer understanding of the study setting and its relevance to the research.	Methods setting section (Line 153-165)
3. The authors state in the limitations that all fathers who	Thank you for this comment, I agree this characteristic should be	Table 2

	participated were in a relationship with or living with the mother of their child. However, this crucial sample characteristic is not reported in the Results section. Please add relationship status (e.g., "living with partner/mother of child") to Table 2 or include it explicitly in the narrative description of the father sample.	included in the sample characteristics table, I have therefore included an extra row entitled Relationship status **"In relationship and co-habiting with mother of child".**	
4.	As the father sample is predominantly White British (12/21) (Results section- Table 2), and all fathers were in a relationship with the mother of their child, this limits the transferability of findings to single fathers, fathers from minority ethnic backgrounds, et al. While the authors acknowledge this limitation, the discussion should more explicitly state how these subgroups' support needs may systematically differ. Future research priorities should be clearly identified.	Thank you for this helpful feedback.<br><br>In response, I have strengthened the Discussion to explicitly acknowledge how the characteristics of the study sample may influence the transferability of the findings. Specifically, I have highlighted that the absence of single fathers and limited representation of ethnically diverse and adoptive fathers restricts the extent to which the findings can be applied to these groups. I have also acknowledged that the support needs and experiences of these fathers may differ from those identified within this study and require further investigation.<br><br>I have expanded the discussion of future research priorities to include exploration of the experiences of adoptive fathers, ethnically diverse and non-English-speaking fathers, and single or widowed fathers. This will help identify how cultural, social and family circumstances may shape fathers' experiences of perinatal support, informing approaches that are both inclusive and responsive to diverse fatherhood experiences.	Strengths and limitations section (Line 697-707)
5.	Inconsistency between inclusion criteria and actual sample： Table 1 lists "NHS professional" as an inclusion criterion for professionals. However, the authors later acknowledge that no health professionals were	Thank you for this helpful feedback.<br><br>In response, I have reviewed the eligibility criteria and removed NHS professionals from the participant criteria, as their	Eligibility criteria Table 1 and limitations section (Line 713-719)

recruited directly through the NHS. This discrepancy should be clarified. Either the inclusion criteria should be revised to reflect the actual sample (VCSE and social care only), or the authors should explain why no NHS professionals participated and how this affects the interpretation of findings related to healthcare service design.	inclusion was not consistent with the intended focus of the study.<br><br>I have also strengthened the Limitations section to acknowledge that the absence of frontline clinical and NHS organisational perspectives may limit the interpretation of findings relating to healthcare service design and implementation. While the study provides important insights into fathers' experiences and support needs, recommendations relating to maternity service delivery would benefit from further exploration with healthcare professionals and service stakeholders to ensure they are feasible, contextually appropriate and aligned with operational realities.	

VERSION 2 - REVIEW

Reviewer 1

Name Tarrant, Anna

Affiliation Lincoln University , School of Social and Political Science

Date 06-Aug-2026

COI

Thank you to the authors for responding effectively to the comments of both reviewers. I am pleased to see this article published.
