# Supplementary material for: Understanding fathers’ perinatal mental health and well-being support needs: a qualitative study of fathers’ and professionals’ perspectives in North East England and North Cumbria, UK
Source: BMJ Open. 2026 Sep 4;16(9):e121953. doi: 10.1136/bmjopen-2026-121953 (PMC13548270; doi:10.1136/bmjopen-2026-121953)
Supplement: Supplementary file 2 [file bmjopen-16-9-s001.docx]

**Interview Topic Guide**

The purpose and focus of this research is to identify what is beneficial for fathers, how fathers can be better supported within the transition to fatherhood and what needs to be improved within paternal perinatal mental health and care from both public and professional perspectives. No comprehensive approach to assessing father’s mental health needs currently exists and with an overwhelming lack of support for fathers within the perinatal period currently it is imperative that research is conducted to understand the experiences of fathers within this period as well as professionals and their opinions on current support.

**Preparation and Safeguarding**

**Instructions for the Interviewer**

Before interview commences thank interviewee for agreeing to participate, introduce myself and who the funder and organisation are.

- Confirm that participant has understood all prior documents.
- Double check consent form is signed correctly
- Explanation of format (what the interview will involve) and approx. length of the interview (60 minutes).
- Go over confidentiality, anonymity of data and how the information will be used within a report as part of a PhD project.
- Remind participant that there is no right or wrong answer and that they do not have to answer every question if they do not want to.
- Emphasise here and again throughout the interview that if the participant chooses to answer any of the questions, they not need to go into depth if they feel it could be a distressing experience.
- Let the participant know now you will have to stop the interview and discuss their information with other members of the team in the case of them disclosing any information which makes us concerned about their safety or the safety of others around them. State that this is our duty of care as researchers so we can support them as best as possible to ensure they are safe.
- Ask participant if they have any further questions.
- Check that participant is happy to continue with interview.
- Lastly check for consent of audio/video recording, if all okay start recording, if not end the interview.

**Interview**

Areas to be covered within interview include experiences of transition into fatherhood, experiences of paternal perinatal mental health support, barrier and facilitators to accessing support and support needs.

**FATHERS INTERVIEW SCHEDULE**

The interview will begin with demographic questions to assess for sample characteristics to form a diverse sample and initial interest in research.

- Can you tell me a little about yourself?
- For example, what is your ethnicity?
- How old are you?
- How long have you been a father?
- How many children do you have/ages of children (experienced or first-time father)?
- Whether you are in education/employment and what this may be?
- What is your educational background?
- What made you interested in taking part in this research?

Following on from answering these questions participant will be asked a series of questions related to exploring the aim of this research as stated above through the use of multiple topics.

**Experiences Transitioning into Fatherhood (Emotional and Social Experiences)**

1. **How did you find the transition to fatherhood/becoming a dad and what was this experience like?**

- What were the most rewarding parts of becoming a dad?
- What was most challenging?
- How prepared did you feel?
- How did you find your mental health and well-being within this period?
- Can you tell me three words that sum up how you felt becoming a dad?

2. **How did you find relationships with others in the transition to fatherhood?**

- How was your relationship with mother of child?
- How was your bond/relationship with child?
- What was the relationship with friends like?
- What was the relationship with parents/in laws like?

**Experiences of Support in the Perinatal Period (Pre-natal and Post-natal)**

3. **Can you tell me about your experiences of support for dads leading up to the birth of your son/daughter?**

- How did you find pre-natal information for dads?
- How did you find educational baby classes if attended?
- How did you find attending appointments before birth?
- How did you find communication from health professionals?
- Did you access any support in the pre-natal period? What type of support did you engage with, for example online support (father groups), through charities/organisations (VCSE) or within the NHS?
- Was it easy to access this type/types of support, or not?
- How did you find support in the pre-natal period?
- Were certain types of support more helpful than others and if so, why?

4. **Can you tell me about your experiences of support for dads after the birth of your son/daughter?**

- How did you find appointments and check-ups after birth?
- What was the communication and support from health professionals like?
- Are you accessing/previously accessed any support within the post-natal period?
- What type of support did you engage with, for example online support (father groups), through charities/organisations (VCSE) or within the NHS?
- Were there any barriers that you found prevented you from accessing support? Which types of support were more accessible?
- How did you find this support? Do you feel it benefitted you or not postnatally?

5. **To gain a better understanding of how we can support dads can you tell me how you feel we can improve support and services concerning mental health or challenges within this period for dads?**

- Pre-natal or post-natal?

**Additional Points/Summary Questions**

6. **Is there anything else that you would like to share?**

- Anything that really helped you?
- Anything that hasn’t helped within the perinatal period and you would change for others?

**PROFESSIONALS INTERVIEW/FOCUS GROUP SCHEDULE**

The interview will begin with demographic questions to assess for sample characteristics (Professional’s role and Organisation) and initial interest in research.

- What made you interested in taking part in this research?
- What comes to mind when we say Paternal Perinatal Mental Health?
- Can you tell me a little about yourself/selves, for example your role or/and association with paternal perinatal mental health support? Do you work within a charitable organisation (VCSE Sector) or within social care or healthcare services? What does your role involve?
- How long have you been working within this role?

Following on from answering these questions participant will be asked a series of questions related to exploring the aim of this research as stated above through the use of multiple topics.

**Experiences of Professional Role Associated with Fathers in the PP**

1. **How have you found working/volunteering within this role associated with either PPMH, PMH or supporting families within the transition to parenthood?**

- What is the most rewarding part about your role?
- What challenges have you encountered within this field of work and why was this the case?
- Do you find many fathers receive support within your organisation/service? Are there any barriers that you are aware of that may prevent fathers from reaching out to your organisation/service?
- How responsive do you consider the support is to the needs of fathers?
- What types of support do you think works best?
- What do you think we could change to make organisations/services more inclusive?
- Can you tell me about your experience of communication between fathers and professionals within your organisation/service or within personal experiences?

**Opinions and Perspectives of Support and Acknowledgement of Fathers in the PP**

2. **What is your perspective and views of PPMH current support?**

- What do you feel would be important for fathers to be informed on or supported with? (education/information/appointments)
- What could be changed within mental health support?
- How do you think fathers would benefit from these changes?
- Imagine if you alone could implement change for fathers within the perinatal period, what would you advise is changed in terms of support, attitudes or in general and how would you implement this change?
- What outcomes could be achieved?

**Additional Points/Summary Questions**

**Is there anything else that you would like to share?**

- Anything that you feel is vitally important relating to PPMH that we have not covered?

**Close for all Interviews/Focus Groups**

- Audio/video recordings stopped.
- Summary of what has been spoken about, thanking participant for taking part and reiterating confidentiality and anonymity.
- Participant will then be provided with a debrief form (hard copy or through email) providing them with further support services if required.
- Participant will be asked if they have any further questions, contact details will also be provided if participant has any queries or concerns or would like to withdraw data after.
- Participant will be assured that online vouchers will be sent out to them via email, if they do not receive it to contact main researcher.
- Ask if once report has been written up if participant would like a copy of the findings and make a note.
